# Pharmacokinetics/pharmacodynamics of ivosidenib in advanced *IDH1*-mutant cholangiocarcinoma: findings from the phase III ClarIDHy study

**DOI:** 10.1007/s00280-023-04633-5

**Published:** 2024-01-27

**Authors:** Bin Fan, Ghassan K. Abou-Alfa, Andrew X. Zhu, Shuchi S. Pandya, Hongxia Jia, Feng Yin, Camelia Gliser, Zhaowei Hua, Mohammad Hossain, Hua Yang

**Affiliations:** 1 Agios Pharmaceuticals Inc., Cambridge, MA USA; 2 Memorial Sloan Kettering Cancer Center, New York, NY USA; 3 Weill Cornell Medicine - Cornell University, New York, NY USA; 4 Trinity College Dublin School of Medicine, Dublin, Ireland; 5 Massachusetts General Cancer Center, Harvard Medical School, Boston, MA USA; 6 Servier Pharmaceuticals LLC, 200 Pier Four Boulevard, Boston, MA 02210 USA; 7 Present Address: Jacobio (US) Pharmaceuticals, Inc., Lexington, MA USA; 8 Present Address: Takeda Pharmaceutical Company Limited, Cambridge, MA USA; 9 Present Address: I-Mab Biophrma, 555 W Haiyang Road New Bund Ctr Fl 55-56, Shanghai, China; 10 Present Address: Pyxis Oncology, Boston, MA USA; 11 Present Address: Disc Medicine, Cambridge, MA USA

**Keywords:** Ivosidenib, Cholangiocarcinoma, Isocitrate dehydrogenase, Pharmacokinetics, Pharmacodynamics, D-2-hydroxyglutarate

## Abstract

**Purpose:**

Report pharmacokinetic (PK)/pharmacodynamic (PD) findings from the phase III ClarIDHy study and any association between PK/PD parameters and treatment outcomes in this population.

**Methods:**

Patients with mutant isocitrate dehydrogenase 1 (m*IDH1*) advanced cholangiocarcinoma were randomized at a 2:1 ratio to receive ivosidenib or matched placebo. Crossover from placebo to ivosidenib was permitted at radiographic disease progression. Blood samples for PK/PD analyses, a secondary endpoint, were collected pre-dose and up to 4 h post-dose on day (D) 1 of cycles (C) 1 − 2, pre-dose and 2 h post-dose on D15 of C1 − 2, and pre-dose on D1 from C3 onwards. Plasma ivosidenib and D-2-hydroxyglutarate (2-HG) were measured using liquid chromatography-tandem mass spectrometry. All clinical responses were centrally reviewed previously.

**Results:**

PK/PD analysis was available for samples from 156 ivosidenib-treated patients. Ivosidenib was absorbed rapidly following single and multiple oral doses (time of maximum observed plasma concentration [*T*_max_] of 2.63 and 2.07 h, respectively). Ivosidenib exposure was higher at C2D1 than after a single dose, with low accumulation. In ivosidenib-treated patients, mean plasma 2-HG concentration was reduced from 1108 ng/mL at baseline to 97.7 ng/mL at C2D1, close to levels previously observed in healthy individuals. An average 2-HG inhibition of 75.0% was observed at steady state. No plasma 2-HG decreases were seen with placebo. Plasma 2-HG reductions were observed in ivosidenib-treated patients irrespective of best overall response (progressive disease, or partial response and stable disease).

**Conclusion:**

Once-daily ivosidenib 500 mg has a favorable PK/PD profile, attesting the 2-HG reduction mechanism of action and, thus, positive outcomes in treated patients with advanced m*IDH1* cholangiocarcinoma.

**Clinical trial registration:**

NCT02989857 Registered February 20, 2017.

**Supplementary Information:**

The online version contains supplementary material available at 10.1007/s00280-023-04633-5.

## Introduction

Intrahepatic cholangiocarcinoma is a rare cancer that has limited treatment options [1]. It is genetically diverse [1–3] and isocitrate dehydrogenase 1 (*IDH1*) mutations have been detected in ~ 13.0% (median, range 8.5–20.0%) of cases [4]. *IDH1* encodes a metabolic enzyme that catalyzes the oxidative decarboxylation of isocitrate to alpha-ketoglutarate (α-KG). In cancer cells, the mutated enzyme reduces α-KG to the oncometabolite D-2-hydroxyglutarate (2-HG) [5, 6], which can be detected in the tumor tissue and blood of patients with cholangiocarcinoma [1, 7]. Research in preclinical models has shown that excessive production and accumulation of 2-HG results in epigenetic and genetic changes that promote tumorigenesis [8–10]. Genetically engineered mouse models expressing mutant IDH in the adult liver show an aberrant response to hepatic injury, consisting of impaired hepatocyte differentiation and elevated levels of cell proliferation [9]. In the same model, mutant IDH and activated Kras (genetic alterations observed in a subset of human intrahepatic cholangiocarcinomas) drives expansion of liver progenitor cells, development of premalignant biliary lesions, and progression to metastatic intrahepatic cholangiocarcinomas [9]. Furthermore, xenografts of cultured mutant IDH2 cells form palpable tumors after subcutaneous injection into mice, whereas vector and wild-type IDH2 cells show no tumorigenicity [8].

Ivosidenib (AG-120) is an oral, potent, targeted inhibitor of the mutant IDH1 (mIDH1) enzyme that is approved for the treatment of patients with locally advanced or metastatic cholangiocarcinoma, and subsets of adult patients with acute myeloid leukemia, with a susceptible *IDH1* mutation [11]. Moreover, ivosidenib has demonstrated favorable pharmacokinetic (PK) and pharmacodynamic (PD) profiles in patients with m*IDH1* solid tumors [12] and m*IDH1* advanced hematologic malignancies [13]. Phase I dose-ranging studies showed good oral exposure after single and multiple doses, rapid absorption, and a long terminal half-life (mean 40–102 h after single dose) [12]. During phase I studies, 500 mg once daily (qd) was determined to be the optimal dose regimen for patients with advanced solid tumors with an IDH1 mutation [12]. No dose-limiting toxicities were reported [14]. In the majority of patients (*n* = 69), even those with progressive disease, plasma 2-HG decreased substantially and persistently and remained at low concentrations [14].

The efficacy and tolerability of ivosidenib in previously treated patients with m*IDH1* advanced cholangiocarcinoma was assessed in the ClarIDHy trial, a phase III, global, multicenter, randomized, double-blind, placebo-controlled study [15]. Progression-free and overall survival, and health-related quality of life, were improved in patients receiving ivosidenib versus placebo, and ivosidenib was well tolerated [15, 16]. This analysis reports the PK/PD findings from ClarIDHy and investigates any association with clinical benefits.

## Methods

### Study design

The study design for ClarIDHy has been described in detail elsewhere [15]. Eligible patients of 18 years of age or older with a histologically confirmed diagnosis of m*IDH1* cholangiocarcinoma were randomized 2:1 to oral ivosidenib 500 mg qd or matched placebo, and stratified by number of prior systemic treatments for advanced disease (one or two). Treatment cycles were 28 (± 2) days long and daily study treatment began on cycle (C) 1 day (D) 1 (C1D1), with continuous dosing. Upon disease progression per investigator assessment, and if the patient continued to meet eligibility criteria, crossover into the ivosidenib treatment arm from placebo was permitted. Patients who crossed over started again with study procedures as at C1D1.

This study was conducted according to the International Council for Harmonisation of Good Clinical Practice guidelines and the principles of the Declaration of Helsinki. Approval from the institutional review board and independent ethics committee was obtained by all study investigators. Informed consent was obtained from all patients included in the study.

### Study assessments and analysis

For radiographic assessments of disease response at baseline and throughout the study period, computed tomography or magnetic resonance imaging were conducted from C1D1 onwards every 6 weeks (± 5 days) through week 48 and every 8 weeks (± 5 days) thereafter, independent of dose delays or interruptions. Objective tumor response was assessed per Response Evaluation Criteria in Solid Tumours version 1.1 (RECIST v1.1) [17] and performed by institutional radiologists. All responses were centrally reviewed by an independent radiology center per RECIST v1.1. Progression-free survival was defined as the time from the date of randomization to the date of first documentation of disease progression or death owing to any cause, whichever occurred first.

On C1D1 (including both C1D1 and crossover C1D1) and C2D1 (including both C2D1 and crossover C2D1), blood samples for PK/PD assessments were drawn pre-dose and at 0.5, 2, and 4 h post-dose. As this a phase III study in patients with a relatively large sample size (*N* = 185), sparse PK sampling over 4-h post-dose was used to capture the maximum concentration (*C*_max_) for concentration-QTc analysis and to perform population PK. On C1D15 and crossover C1D15, blood samples were drawn pre-dose and at 2 h (± 10 min) post-dose. On C3D1 and crossover C3D1 and D1 of each treatment cycle thereafter (including crossover), and at any time during the end-of-treatment visit, blood samples were drawn pre-dose (within 30 min).

Plasma ivosidenib was measured using a validated liquid chromatography-tandem mass spectrometry (LC–MS/MS) method, with a lower limit of quantitation (LLOQ) of 50.0 ng/mL. Plasma 2-HG concentrations were measured using a qualified LC–MS/MS method, with a LLOQ of 29.6 ng/mL.

Data analysis and processing were completed using a validated version of Phoenix^®^ WinNonlin^®^ 7.0 (Certara, Princeton, NJ) or R v3.3.1 (R Core Team, Vienna, Austria). Additional graphing was performed with Prism 9.4.1 (GraphPad Software, Boston, MA). Enrolled patients who received at least one dose of ivosidenib and who had sufficient plasma sample data to assess PK or PD parameters comprised the PK or PD analysis populations. The PK/PD analysis population included all patients in the PK analysis population who had at least one PD concentration data point time-matched to the PK concentration.

### PK/PD methods

Concentration values of plasma ivosidenib reported as below the limit of quantitation (BLQ) were set to 0 for the PK and statistical analyses. Plasma 2-HG levels reported as BLQ were set to the value of the LLOQ for the PD and statistical analyses. All plasma PK parameter calculations were performed using actual times calculated relative to the most recent time of study drug administration. PK parameters were determined using non-compartmental analysis (NCA) methods based on individual plasma concentration–time data for ivosidenib and included:

- Time of the last quantifiable concentration (*T*_last_)
- Area under the plasma concentration–time curve from time 0 to 4 h (AUC_0–4_)
- Area under the plasma concentration–time curve from time 0 to *T*_last_ (AUC_0–*t*_)
- Maximum observed plasma concentration (*C*_max_)
- Time of maximum plasma concentration (*T*_max_)
- Observed concentration at the end of a dosing interval, right before the next dose (*C*_trough_)
- Accumulation ratio based on *C*_max_ (*R*_acc_ (*C*_max_)), calculated as:
  $${R}_{{\text{acc}}} ({C}_{{\text{max}}})=\frac{{C}_{{\text{max}},\,\mathrm{ steady}-\mathrm{state }}}{{C}_{{\text{max}},\,\mathrm{ single dose}}}$$
- Accumulation ratio based on AUC_0–4_ (*R*_acc_ (AUC_0–4_)), calculated as:
  $${R}_{{\text{acc}}} ({{\text{AUC}}}_{0-4})=\frac{{{\text{AUC}}}_{0-4,\,\mathrm{ steady}-\mathrm{state }}}{{{\text{AUC}}}_{0-4,\,\mathrm{ single dose}}}$$

Actual doses of ivosidenib were used to calculate PK parameters, and concentration–time profiles were excluded from the analyses for all missed doses or dose adjustments. Data interpolation was applied for any missing plasma concentration at the end time of the pre-defined AUC time curve. If the pre-defined end time fell within the range of the available data but did not coincide with an observed data point, then the plasma concentration corresponding to the missing time point was estimated (imputed) by performing a linear interpolation, as applicable. If the pre-defined end time fell outside the range of the available data (i.e., if the last available data for calculation of AUC_0–4_ was 3.98 h), one of the following two methods was used: (1) if the 4-h sample was collected within 48 min prior to 4 h post-dose (i.e., 20% deviation from the scheduled 4 h allowed) AUC_0–*t*_ was used to estimate AUC_0–4_; (2) if the 4-h sample was collected more than 48 min prior to 4 h, AUC_0–4_ was not reported. Observed individual plasma concentrations and percent inhibition of 2-HG were analyzed using actual times (nominal times were used when actual times were not available) and calculated relative to the most recent time of study drug administration.

Patient-specific plasma 2-HG values at baseline were used for baseline adjustment. PD parameters were determined using NCA methods, based on individual observed plasma concentration–time data for 2-HG, and included:

- The last non-missing observation collected from each patient before the first ivosidenib dose (baseline effect value, *B*)
- Area of the response curve from time point 0 (pre-dose) up to 4 h post-dose (AUEC_0–4_)
- Percent inhibition for AUEC_0–4_ (%BAUEC_0–4_), calculated as:
  $$ {\text{\% BAUEC}}_{0 - 4} { = }\left( {\frac{{\left[ {B \times T_{{4\;{\text{hour}}}} } \right] - {\text{AUEC}}_{0 - 4} }}{{\left[ {B \times T_{{4\;{\text{hour}}}} } \right]}}} \right) \times 100 $$
- Observed response value at the end of a dosing interval immediately before the next dose (*R*_trough_)
- Percent inhibition for *R*_trough_ (%BR_trough_), calculated as:
  $${\mathrm{\%BR}}_{{\text{trough}}}=\left(\frac{B-{R}_{{\text{trough}}}}{B}\right)\times 100$$

### PK/PD correlation analysis

Correlations between selected plasma ivosidenib PK parameters and selected plasma 2-HG PD parameters at C2D1 were explored using graphical display of data. The strength of the PK/PD relationships was assessed using locally weighted scatterplot smoothing. Longitudinal PK/PD profiles of pre-dose plasma ivosidenib and plasma 2-HG were also derived.

Associations between steady-state plasma 2-HG and clinical response were also assessed.

### Statistical analysis

Individual plasma PK and PD parameters were listed and summarized in accordance with grouping factors (i.e., cycle and day). For all summary statistics, crossover C1D1 and crossover C2D1 visits were combined with C1D1 and C2D1 visits, respectively. Descriptive statistics were used to summarize concentration data at each nominal time point.

## Results

### Patients and data sets analysis

Patient recruitment occurred between February 20, 2017 and March 1, 2019. Enrollment has completed, with 187 patients randomized to either ivosidenib (*n* = 126) or placebo (*n* = 61); the study completed in May 2021. Data included in this manuscript are from the primary endpoint analysis with a data cut-off of January 31, 2019. As of this date, 185 patients were enrolled: 124 randomized to ivosidenib and 61 to placebo. Patient demographic and disease characteristics are summarized in Table S1 of the Supplementary Information.

**Fig. 1:**
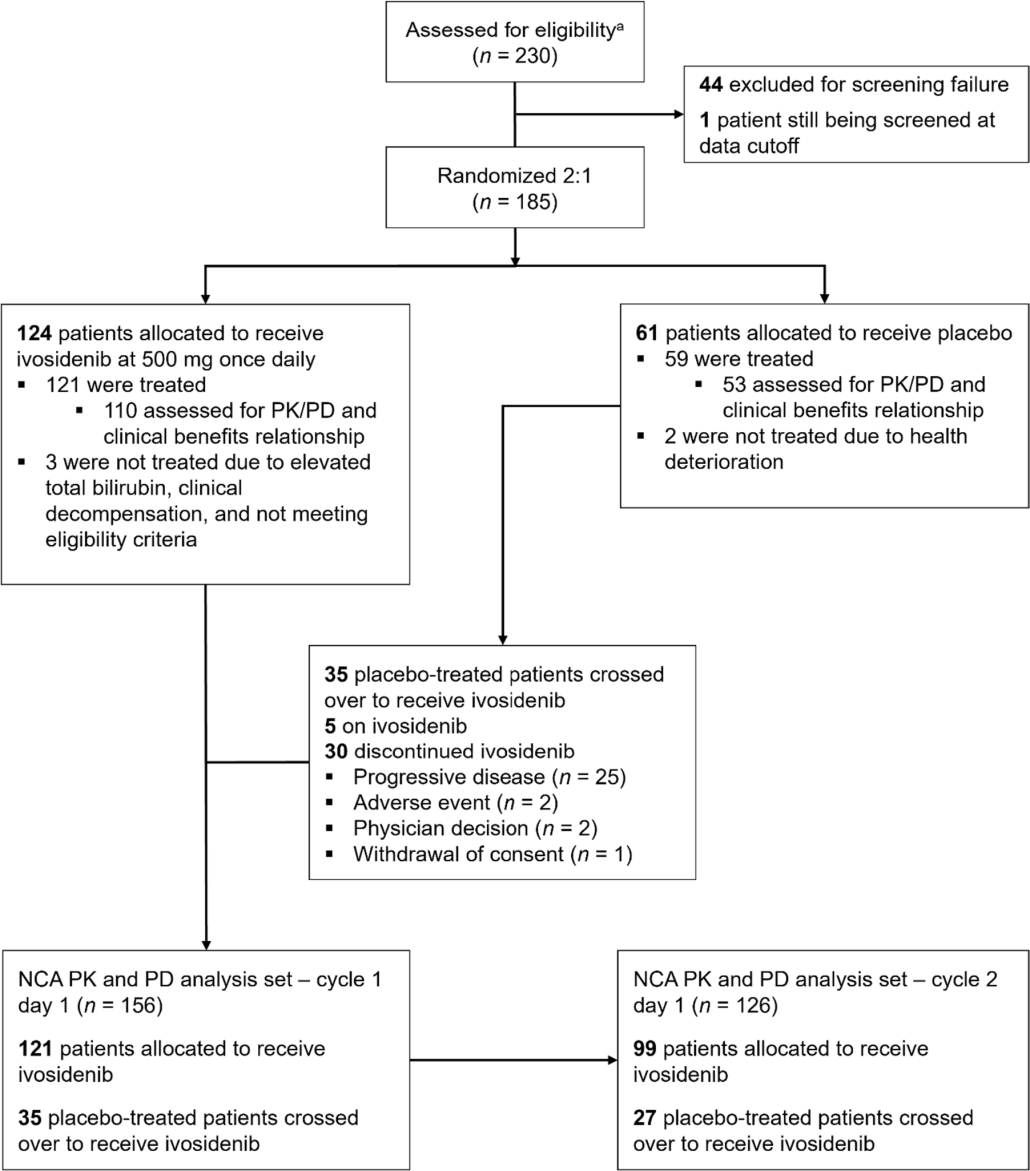
CONSORT diagram. ^a^As of data cut-off, January 31, 2019. *NCA* non-compartmental analysis, *PD* pharmacodynamics, *PK* pharmacokinetic

For the PK and PD analyses, samples were analyzed from 156 patients who received ivosidenib at the C1D1 visit (including 121 patients initially assigned to ivosidenib and 35 patients who crossed over from placebo), and from 126 patients who received ivosidenib at the C2D1 visit (99 active ivosidenib-treated patients and 27 crossover patients) (Fig. 1). Overall, samples from 105 patients who received ivosidenib and 57 patients who received placebo were analyzed to assess any relationship between 2-HG levels and clinical response.

### PK analysis

**Fig. 2:**
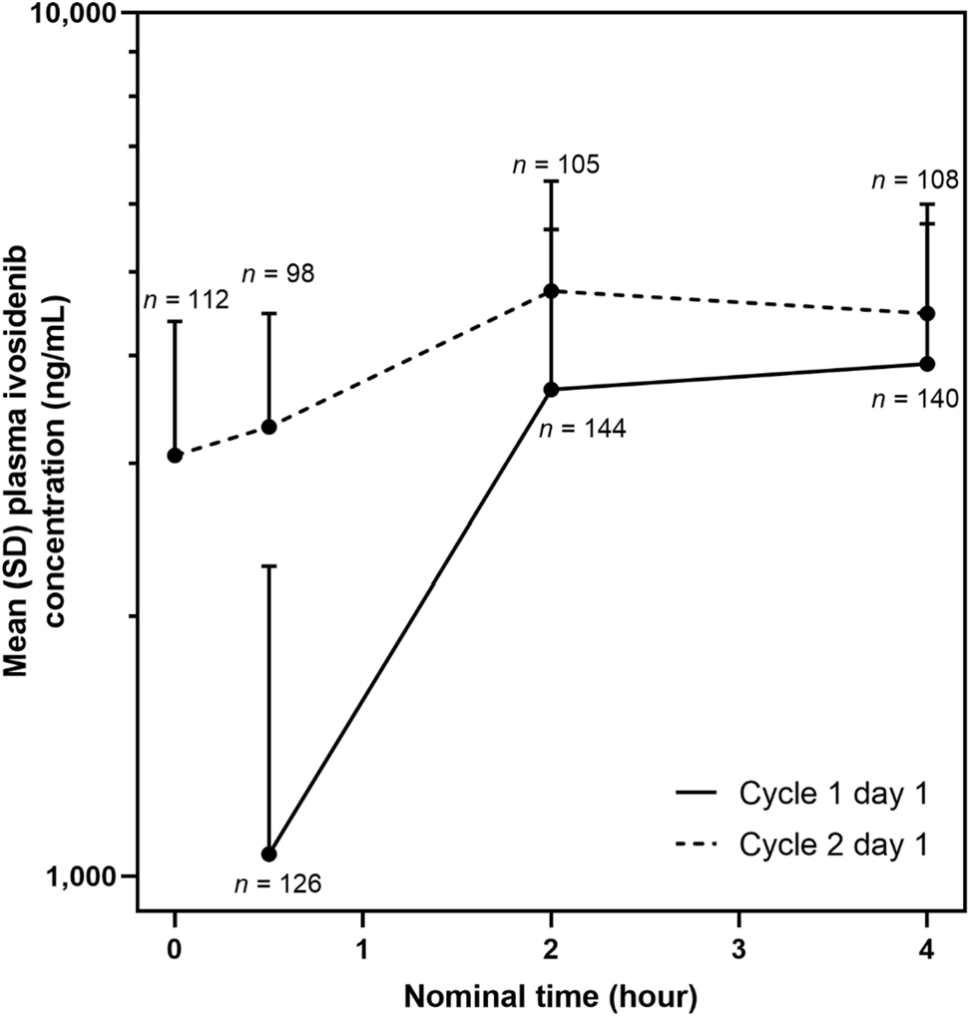
Mean (SD) plasma concentration over time after oral administration of ivosidenib 500 mg once daily

Ivosidenib was absorbed rapidly following single (median *T*_max_, 2.63 [0.5–4.87] h) and multiple oral doses (median *T*_max_, 2.07 [0.50–4.08] h; Table 1, Fig. 2). Ivosidenib exposure, measured by *C*_max_ and AUC_0–4_, was higher after multiple doses than after a single dose, with some accumulation (geometric mean accumulation ratios of 1.16 and 1.54 for *C*_max_ and AUC_0–4_, respectively). The geometric mean plasma ivosidenib *C*_max_ was 4060 ng/mL after a single dose versus 4799 ng/mL following multiple doses (500 mg qd). After a single dose, the geometric mean plasma ivosidenib AUC_0–4_ was 9760 h·ng/mL versus 15,887 h·ng/mL after multiple doses. After multiple doses, the geometric mean plasma ivosidenib AUC_0–24_ was 86,382 h·ng/mL. Plasma ivosidenib levels remained constant from C1 to the end of the study and appeared to reach steady state during the first cycle of continuous dosing.

**Table 1:** Summary of plasma PK parameters after single and multiple oral doses of ivosidenib 500 mg once daily

Visit	PK parameter^a^	Patients, *n*	Geometric mean	GeoCV%
C1D1	AUC_0–4_, h·ng/mL	141	9760	55.4
	*C*_max_, ng/mL	142	4060	45.4
	*T*_max_, h^b^	142	2.63	(0.50, 4.87^d^)
C2D1	AUC_0–4_, h·ng/mL	106	15,887	31.5
	AUC_0–24_, h·ng/mL^c^	107	86,382	33.8
	*C*_max_, ng/mL	107	4799	32.9
	*T*_max_, h^b^	107	2.07	(0.50, 4.08)
	*R*_acc_ (AUC_0–4_)	98	1.54	42.9
	*R*_acc_ (*C*_max_)	100	1.16	37.2

*AUC*_*0–x*_ area under the plasma concentration–time curve from time 0 to *x* h, *C* cycle, *C*_*max*_ maximum observed plasma concentration, *D* day, *GeoCV%* geometric coefficient of variation, *PK* pharmacokinetic, *R*_*acc*_* (AUC*_*0–4*_*)* accumulation ratio based on area under the plasma concentration–time curve from time 0 to 4 h, *R*_*acc*_* (C*_*max*_*)* accumulation ratio based on maximum observed plasma concentration, *T*_*max*_ time of maximum observed plasma concentration

^a^Geometric mean (GeoCV%), unless otherwise specified

^b^Median (minimum, maximum)

^c^For the calculation of AUC_0–24_, the plasma concentration corresponding to the nominal 24 h at C2D1 was imputed using the pre-dose concentration at C2D1

^d^As the *T*_max_ value of 4.87 h for one subject on C1D1 has more than 20% deviation from the scheduled time of 4 h, it was excluded from the summary statistics

### PD analysis

Plasma 2-HG concentrations decreased for the duration of the observation period compared with 2-HG concentrations recorded at baseline (Table 2). Following single and multiple doses of ivosidenib (500 mg qd), the mean plasma 2-HG AUC_0–4_ was 3334 h·ng/mL and 368 h·ng/mL, respectively. The mean plasma 2-HG concentration decreased from 1108 ng/mL at baseline to 97.7 ng/mL at C2D1, close to levels observed in healthy individuals (72.6 ± 21.8 ng/mL) [12]. Following a single dose of ivosidenib (500 mg qd), the average 2-HG inhibition (based on %BAUC_0-4_) was 20.2%. An average 2-HG inhibition of 75.0% (up to 97.3%) was observed at steady state after multiple ivosidenib administrations (Fig. S1, Supplementary Information). The observed 2-HG inhibition was robust and persisted up to C19. Changes in plasma 2-HG concentrations based on AUEC_0–4_ by visit are presented in Figures S2 and S3 of the Supplementary Information. Among the 5 allele types (R132C/L/G/H/S) tested, 70% were R132C, while only 15% were R132L, 12% were R132G, and < 2% were R132H/S. The relationship between IDH1 mutation isotype and 2-HG inhibition was not investigated in this study, as a previous phase 1 study showed the median values of 2-HG inhibition based on AUC were comparable between the different isotypes in subjects with cholangiocarcinoma [12].

**Table 2:** Mean plasma PD parameters of D-2-hydroxyglutarate after single and multiple oral doses of ivosidenib 500 mg once daily

Visit	PD parameter	Patients, *n*	Mean	CV%
C1D1	*B*, ng/mL	142	1108	154.4
	AUEC_0–4_, h·ng/mL	141	3334	143.5
	%BAUEC_0–4_, %	141	20.2	50.1
C2D1	AUEC_0–4_, h·ng/mL	107	368	75.7
	%BAUEC_0–4_, %	107	75.0	30.1
	*R*_trough_, ng/mL	108	97.7	74.6
	%BR_trough_, %	108	73.7	31.6

*%BAUEC*_*0–4*_ percent inhibition for area of the response curve from time point 0 (pre-dose) up to 4 h post-dose, *%BR*_*trough*_ percent inhibition for observed response value at the end of a dosing interval, *AUEC*_*0–4*_ area of the response curve from time point 0 (pre-dose) up to 4 h post-dose, *B* baseline effect value, *C* cycle, *CV%* coefficient of variation, *D* day, *PD* pharmacodynamics, *R*_*trough*_ observed response value at the end of a dosing interval

### PK/PD correlation

**Fig. 3:**
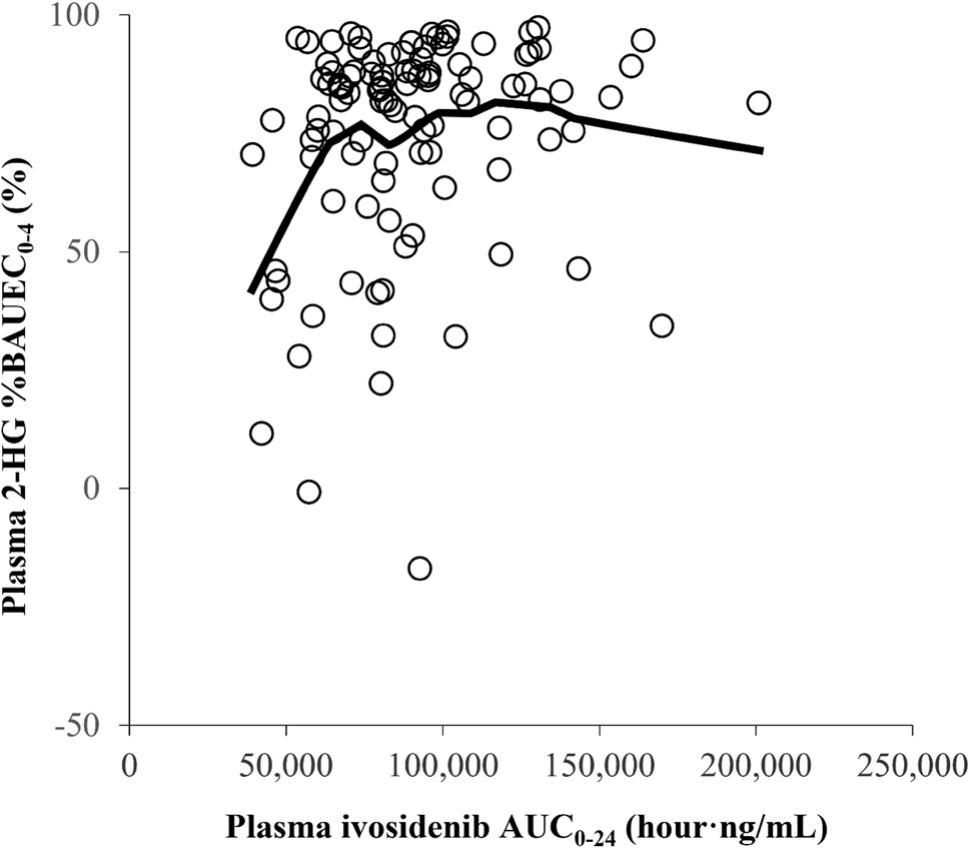
Scatter plot of D-2-hydroxyglutarate (2-HG) percent inhibition versus plasma ivosidenib after multiple doses at cycle 2 day 1 (ivosidenib 500 mg once daily). %BAUEC_0–4_, percent inhibition for area under the effect concentration–time curve from pre-dose up to 4 h post-dose; AUC_0–24_, area under the plasma concentration–time curve from time point 0 (pre-dose) up to 24 h post-dose

The analysis of longitudinal PK/PD profiles during the observation period indicated that plasma ivosidenib reached steady state during the first cycle after multiple doses of 500 mg ivosidenib and was associated with decreasing levels of 2-HG to values observed in healthy individuals (Fig. S4). In most patients, plasma 2-HG levels were reduced by > 50% with the daily dose of ivosidenib 500 mg relative to the 2-HG levels observed at baseline. After daily ivosidenib 500 mg dosing, plasma 2-HG percent suppression reached > 70% over the majority (approximately 60,000–200,000 h.ng/mL) of the observed range of plasma ivosidenib exposure at C2D1 (Fig. 3). No relationship was observed between exposure (AUC or *C*_max_) and clinical response. In this phase III study, only 500 mg QD dose was evaluated and at this dose the %2-HG reduction observed was similar for steady-state *C*_max_, *C*_min_, or AUC. The suppression of 2-HG following ivosidenib administration was maintained throughout the treatment period (Fig. S4, Supplementary Information).

### Plasma 2-HG levels and treatment outcome

**Fig. 4:**
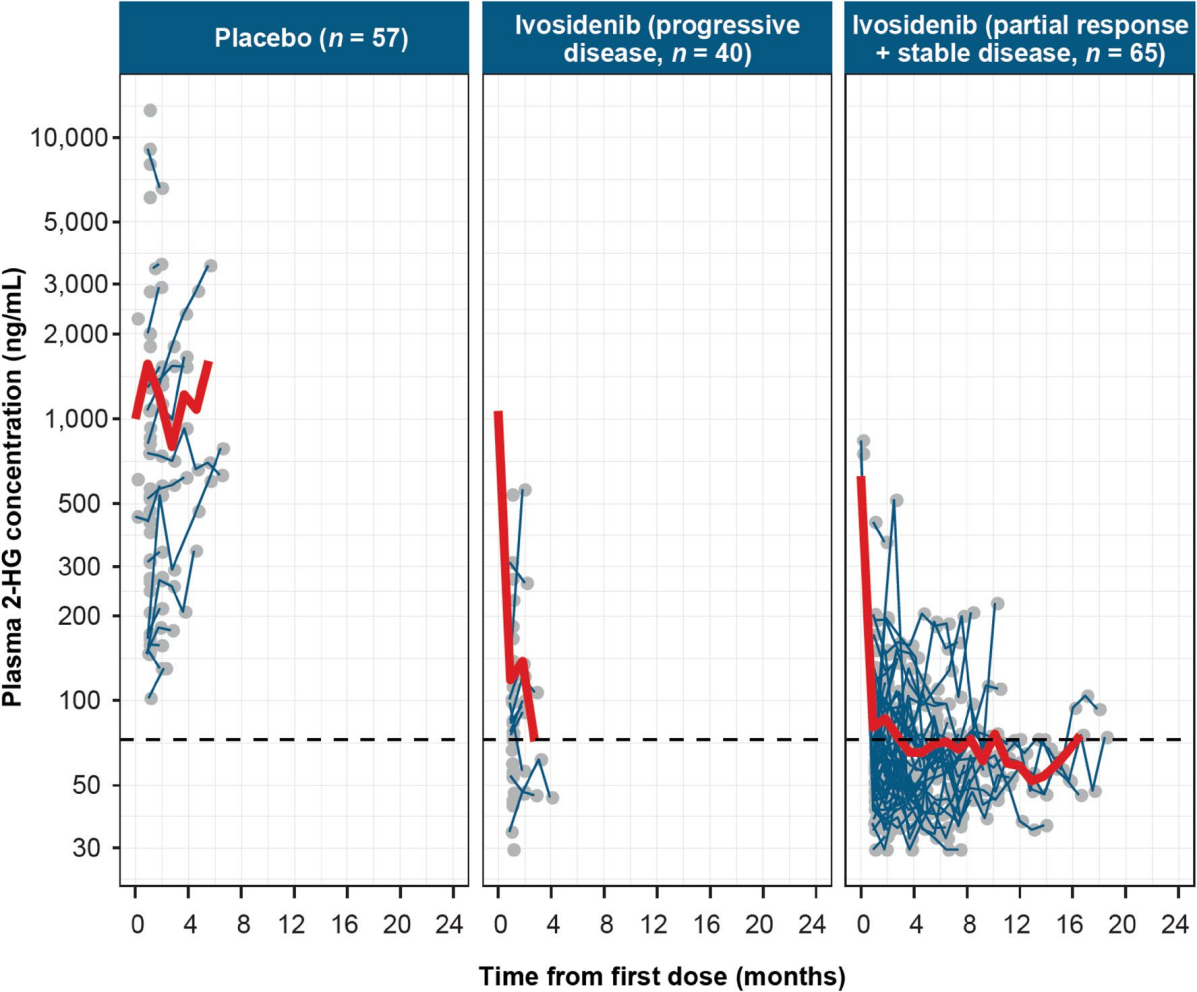
Plasma D-2-hydroxyglutarate (2-HG) profile in placebo arm and ivosidenib treatment arm. Gray circles represent observed data. Each blue line represents one patient. Red bold line represents the arithmetic mean of the observed data for each time point (when *n* ≥ 3). Dashed line represents the plasma 2-HG level (72.6 ng/mL) in healthy individuals

The distribution profiles of plasma 2-HG levels by best overall response (partial response plus stable disease [*n* = 65]; progressive disease [*n* = 40]) for patients receiving ivosidenib compared with those receiving placebo (*n* = 57) are shown in Fig. 4. Although plasma 2-HG concentrations in patients receiving placebo remained elevated and increased relative to baseline over the observation period, plasma 2-HG concentrations in patients who achieved a best overall response of partial response or stable disease on ivosidenib decreased to levels observed in healthy individuals during the first cycle of dosing and remained stable over the observation period. A similar trend was seen in patients who achieved a best overall response of progressive disease, with plasma 2-HG concentrations also decreasing to close to levels observed in healthy individuals.

## Discussion

Ivosidenib is a potent and targeted inhibitor of mIDH1 and was shown to reduce plasma 2-HG levels substantially in patients with solid tumors and hematologic malignancies [12, 13]. In the phase III ClarIDHy study, ivosidenib demonstrated an improvement in progression-free survival compared with placebo in patients with advanced previously treated m*IDH1* cholangiocarcinoma [15]. The results from this study align with previously reported pharmacokinetic parameters of ivosidenib in patients with intrahepatic cholangiocarcinoma [18]. In this report, oral ivosidenib 500 mg qd demonstrated good exposure in patients with advanced m*IDH1* cholangiocarcinoma. Plasma ivosidenib exposure in this population following single or multiple doses of ivosidenib was comparable with findings from a phase I study of patients with advanced solid tumors, including cholangiocarcinoma [12]. Additionally, and as reported in the aforementioned phase I study [12], following one cycle of ivosidenib, mean plasma 2-HG concentration in this population was reduced by up to ~ 97%, close to levels observed in healthy individuals (72.6 ± 21.8 ng/mL) [12]. Plasma 2-HG inhibition was generally maintained through the observation period with continuous dosing of ivosidenib 500 mg qd.

Ivosidenib was rapidly absorbed following single and multiple qd 500 mg doses. Moreover, plasma ivosidenib levels appeared to reach steady state during the first cycle of continuous dosing, consistent with the recently published findings in solid tumors, including cholangiocarcinoma [12]. Ivosidenib 500 mg qd was associated with a robust and persistent reduction of plasma 2-HG, irrespective of treatment outcome. The allowance of placebo-to-ivosidenib crossover in the trial did not limit the PK/PD analysis reported here. However, this analysis provided only limited characterization of any associations between 2-HG reduction and clinical outcomes. A range of disease- and host-specific factors, such as the impact of tumor burden on 2-HG production and/or reduction, may influence clinical response to mIDH1 inhibition, and these covariates were not assessed here. Furthermore, paired tissue biopsies were not taken in this study, therefore no analyses could be conducted with regard to resistance dynamics and mechanisms. Potentially, many molecular factors may contribute to the differences in treatment outcomes seen with 2-HG reduction. Previous analysis of matched baseline and on-treatment samples from patients with mIDH1 intrahepatic cholangiocarcinoma treated in the phase I study of ivosidenib in patients with solid tumors showed that ivosidenib induced morphological changes (notably, decreased cytoplasm) and molecular evidence of hepatocytic differentiation, both of which were correlated with improved progression-free survival [19]. Conversely, earlier disease progression was associated with AKT activity, cell proliferation, and stem cell gene expression signatures. Ongoing exploratory efforts, including analyses of liquid biopsies, may provide further elucidation of any relationship between plasma 2-HG reduction and clinical response in ClarIDHy.

In conclusion, ivosidenib 500 mg qd has shown a favorable PK/PD profile in patients with advanced m*IDH1* cholangiocarcinoma, with demonstrated rapid absorption, slow elimination, and robust suppression of plasma 2-HG, attesting the positive outcomes in treated patients.

### Supplementary Information

Below is the link to the electronic supplementary material.

Supplementary file1 (DOCX 177 KB)

## Data Availability

Study-level clinical data from this study (including the protocol) will be made available upon reasonable request from a qualified medical or scientific professional for the specific purpose laid out in that request and may include deidentified individual participant data. The data for this request will be available after a data access agreement has been signed. Please send your data sharing request to https://clinicaltrials.servier.com/data-request-portal/.
