# Supplementary material for: Pharmacokinetics/pharmacodynamics of ivosidenib in advanced IDH1-mutant cholangiocarcinoma: findings from the phase III ClarIDHy study
Source: Cancer Chemother Pharmacol. 2024 Jan 27;93(5):471–9. doi: 10.1007/s00280-023-04633-5 (PMC11043204; doi:10.1007/s00280-023-04633-5)
Supplement: Supplementary file 1 — Supplementary file1 (DOCX 177 KB) [file 280_2023_4633_MOESM1_ESM.docx]

**Pharmacokinetics/pharmacodynamics of ivosidenib in advanced *IDH1*-mutant cholangiocarcinoma: findings from the phase III ClarIDHy study**

Bin Fan, Ghassan K. Abou-Alfa, Andrew X. Zhu, Shuchi S. Pandya, Hongxia Jia, Feng Yin, Camelia Gliser, Zhaowei Hua, Mohammad Hossain and Hua Yang

**Correspondence:** Mohammad Hossain, Servier Pharmaceuticals LLC, 200 Pier Four Boulevard, Boston, MA, 02210, USA; Tel: +1(267) 853-3860 ([mohammad.hossain@servier.com](mailto:mohammad.hossain@servier.com)), ORCID 0000-0002-9147-7917

**Journal:** Cancer Chemotherapy and Pharmacology

**SUPPLEMENTARY INFORMATION**

**SUPPLEMENTAL FIGURES AND TABLES**

**Fig. S1** Mean D-2-hydroxyglutarate ([a] observed concentrations and [b] percent inhibition) over time after oral administration of ivosidenib 500 mg once daily. 2-HG, D-2-hydroxyglutarate

**
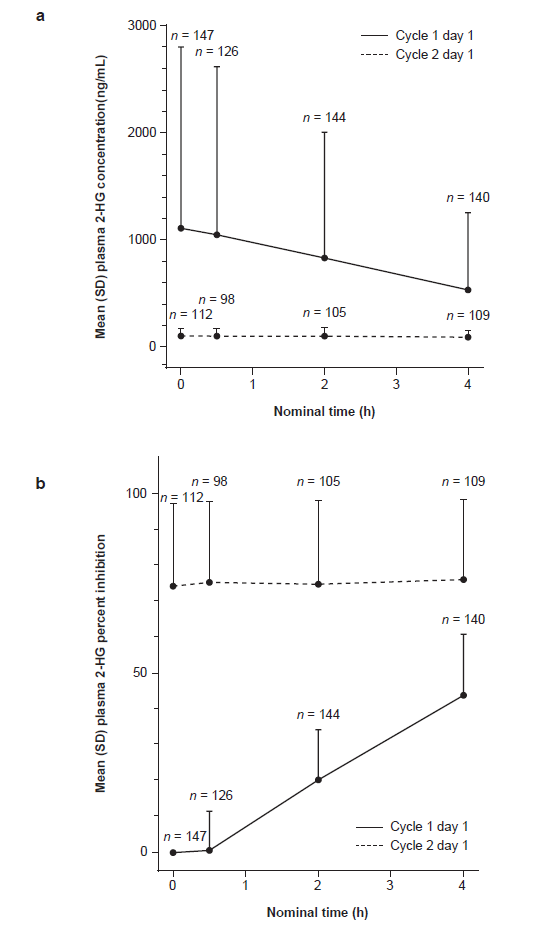
**

## **Fig. S2** Plasma D-2-hydroxyglutarate (2-HG) levels based on area under the effect concentration-time curve from time point 0 (pre-dose) up to 4 h post-dose (AUEC_0–4_) by visit (ivosidenib 500 mg once daily). Boxes denote 25th and 75th percentiles, lines denote the median, and whiskers range from minimum to maximum. Numbers above the boxes refer to data count at each visit


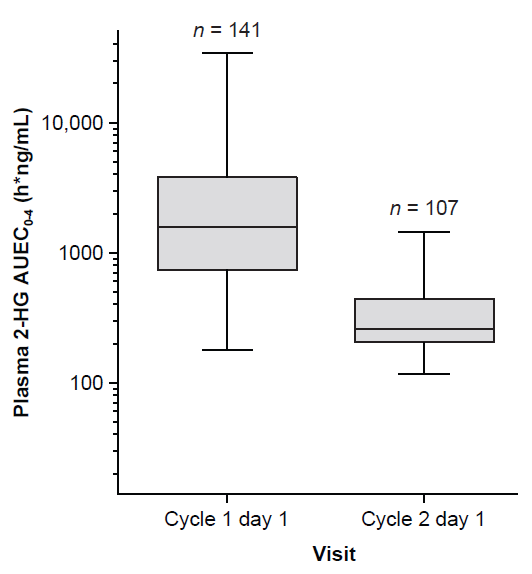


## **Fig. S3** Percent inhibition of plasma D-2-hydroxyglutarate (2-HG) based on percent inhibition for area under the effect concentration-time curve from pre-dose up to 4 h post-dose (%BAUEC_0–4_) by visit (ivosidenib 500 mg once daily). Lines denote the median; boxes, whiskers, and the solid black circles are plotted with Tukey’s method. Numbers above the boxes refer to data count at each visit


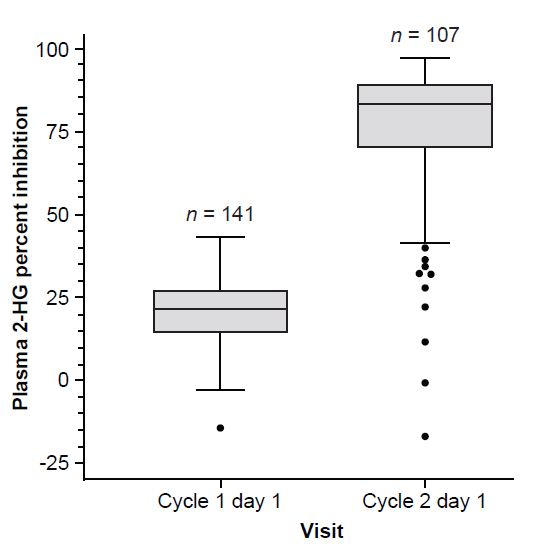


## **Fig. S4** Longitudinal profiles of pre-dose (trough) plasma ivosidenib and pre-dose (trough) plasma D-2-hydroxyglutarate (2-HG) after oral administration of ivosidenib (500 mg once daily). Lines denote the median; boxes, whiskers, and the solid black circles are plotted with Tukey’s method. Numbers below the box plot present data count at each visit. The dashed line in the top panel represents the mean 2-HG baseline in healthy individuals (72.6 ng/mL) [1]. C, cycle; D, day


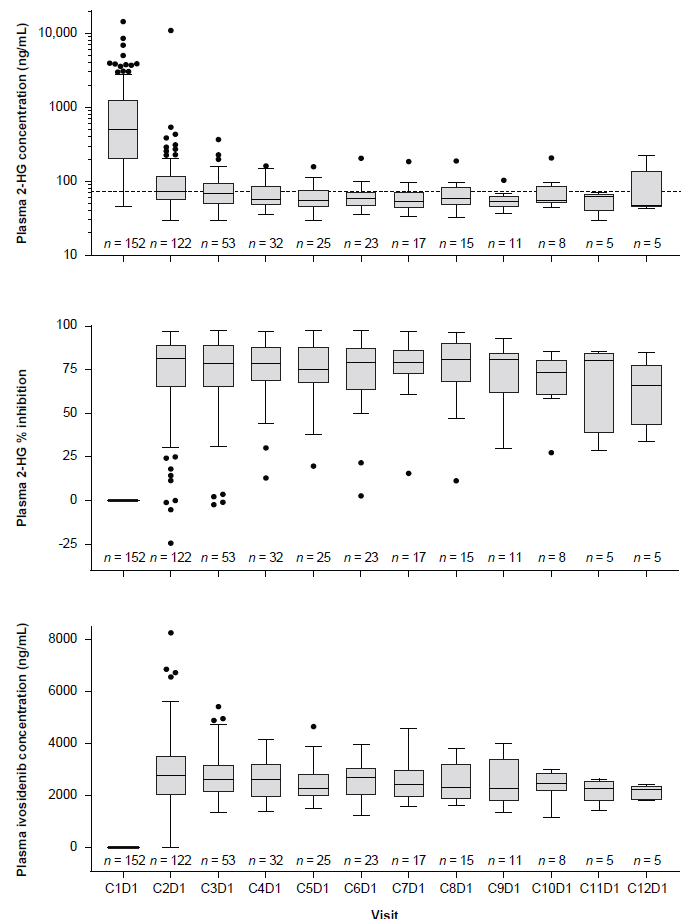


## **Table S1** Demographic and baseline characteristics recorded on January 31, 2019

| **Characteristic** | **Ivosidenib**  **(*n* = 124)** | **Placebo**  **(*n* = 61)** |
| --- | --- | --- |
| Female/male, *n* | 80/44 | 37/24 |
| Age, median (range), years | 61 (33–80) | 63 (40–83) |
| Randomization strata, *n* (%) |  |  |
| 1 prior line of therapy | 66 (53.2) | 33 (54.1) |
| 2 prior lines of therapy | 58 (46.8) | 28 (45.9) |
| ECOG status at baseline, *n* (%) |  |  |
| 0 | 49 (39.5) | 19 (31.1) |
| 1 | 74 (59.7) | 41 (67.2) |
| 2 | 0 | 1 (1.6) |
| 3 | 1 (0.8) | 0 |
| Cholangiocarcinoma type at diagnosis, *n* (%) |  |  |
| Intrahepatic | 111 (89.5) | 58 (95.1) |
| Extrahepatic | 1 (0.8) | 1 (1.6) |
| Perihilar | 4 (3.2) | 0 |
| Unknown | 8 (6.5) | 2 (3.3) |
| Extent of disease at screening, *n* (%) |  |  |
| Local/regional | 9 (7.3) | 5 (8.2) |
| Metastatic | 115 (92.7) | 56 (91.8) |

ECOG, Eastern Cooperative Oncology Group.

[Table adapted from Abou-Alfa, G.K., et al., Ivosidenib in IDH1-mutant, chemotherapy-refractory cholangiocarcinoma (ClarIDHy): a multicentre, randomised, double-blind, placebo-controlled, phase 3 study. Lancet Oncol, 2020. 21(6): p. 796-807. © 2020 Elsevier Ltd.]

**References**

1. Fan B et al. Clinical pharmacokinetics and pharmacodynamics of ivosidenib, an oral, targeted inhibitor of mutant IDH1, in patients with advanced solid tumors (2020) Invest New Drugs 38:433–444.
